# Physicochemical and Immunomodulatory Properties of Gum Exudates Obtained from *Astragalus myriacanthus* and Some of Its Isolated Carbohydrate Biopolymers

**Published:** 2017

**Authors:** Azadeh Hamedi, Gholamhossein Yousefi, Shirin Farjadian, Mitra Saadat Bour Bour, Elahenaz Parhizkar

**Affiliations:** a *Medicinal Plants Processing Research Center, Shiraz University of Medical Sciences, Shiraz, Iran.*; b *Department of Pharmacognosy, School of Pharmacy, Shiraz University of Medical Sciences, Shiraz, Iran.*; c *Department of Pharmaceutics, School of Pharmacy, Shiraz University of Medical Sciences, Shiraz, Iran.*; d *Center for Nanotechnology in Drug delivery, Shiraz University of Medical Sciences, Shiraz, Iran. *; e *Department of Immunology, School of Medicine, Shiraz University of Medical Sciences, Shiraz, Iran. *; f *Student Research Committee, School of Pharmacy, Shiraz University of Medical Sciences, Shiraz, Iran.*

**Keywords:** Astracantha myriacantha, Mucoadhesive, Cytotoxicity, Gum, Water Soluble Polysaccharide, Immunomodulatory, carbohydrate

## Abstract

Plants gums are complex mixtures of different polysaccharides with a variety of biological activities and pharmaceutical applications. Few studies have focused on physicochemical and biological properties of gums obtained from different plants.

This study was designed to determine potential pharmaceutical and pharmacological values of the gum exudates and its isolated biopolymers obtained from *Astragalus myriacanthus *Boiss [syn. *Astracantha myriacantha *(Boiss.) Podlech] (Fabaceae).

The physicochemical, rheological, and mucoadhesion properties of the gum and its fractions was measured at 7, 27, and 37 °C with and without the presence of NaCl (1%). Also, the structural and immunomodulatory properties of several water soluble biopolymers isolated using ion exchange and size exclusion chromatographic methods were investigated on Jurkat cells at concentrations of 31.25, 62.5, 125, 250, 500 and 1000 μg/mL.

The consistency and shear-thinning property of the gum and its fractions decreased as temperature increased. In the presence of NaCl, the consistency increased but no regular pattern was observed regarding to shear-thinning behavior. The mucoadhesion strength was 40.66 ± 2.08 g/cm^2^ which is suitable for use as a formulary mucoadhesive polymer. The isolated biopolymers had proteo-arabinoglycan structure. Their molecular weight was calculated to be 1.67-667 kDa. One biopolymer had a proliferative effect and others had dose dependent cytotoxic/proliferative properties. The crude gum and its insoluble fraction showed suitable mucoadhesion, swellability and rheological properties which makes them suitable for designing drug delivery systems. The gum proteo-arabinoglycans with different molecular weight and structures had different immunomodulatory properties.

## Introduction

*Astragalus* genus contains 1500 species (1). The gum obtained from some of its species has a wide variety of applications in the pharmaceutical (2), cosmetic (3) and food industries (4-6), mostly for its gel production properties. Little study has focused on the physicochemical and biological properties of its polysaccharides; especially that the gums which are available in herbal market are mixture of exudates obtained from different species. Problems associated with gum tragacanth standardization stems from the variation in rheological and chemical characteristics that change depending on the species used, and the geographical and seasonal characteristics and harvesting mode (7). It is important to determine the physicochemical properties of gum exudates obtained from different species of *Astragalus* genus to improve its industrial standardization and application. 

Beside pharmaceutical applications of gums as formulary excipients, the polysaccharides of these gums may have potential biological activities especially that many biological properties have been reported for polysaccharides of different sources, including specific and non-specific immunomodulatory (8, 9), antioxidant (10), anticoagulant (11), hypocholesterolemic (12), antiviral (13), anti-inflammatory (14, 15), keratinocytes DNA repair (16), and antitumor (17, 18) properties, but not all polysaccharides exhibit these effects. Of the biological activities of polysaccharides, their antitumor and immunomodulatory properties are a focus of interest for researchers. 

This study was designed to comprehensively investigate formulary pharmaceutical characteristics (swelling, rheology and mucoadhesion behaviors), pharmacological potential values (immunomodulatory properties of soluble isolated biopolymers), and physicochemical properties of gum exudates obtained from *Astragalus myriacanthus *Boiss [syn. *Astracantha myriacantha *(Boiss.) Podlech] (Fabaceae) and its isolated biopolymers.

## Experimental

*Materials*

Gum exudate of *A. myriacanthus* was collected in September 2014 from plants growing in the central mountains of Fars province in Iran. The plant was authenticated by Parviz Zandi (a taxonomist at Agricultural and Natural Resources Research Center, Zarghan, Iran) and its herbarium voucher specimen (code 775-1) is on file at the Department of Pharmacognosy of Shiraz University of Medical Sciences, Shiraz, Iran.

Human Jurkat E6.1 cells (NCBI code: C121, Jurkat-FHCRC cell line) was purchased from Pasteur Institute of Iran. The cell proliferation reagent WST-1 (sodium salt of 4-[3-(4iodophenyl)-2-(4-nitrophenyl)-2H-5-tetrazolio]-1, 3-benzene disulfonate) was purchased from Roche Applied Sciences, Germany. Other materials, reagents, and solvents were purchased from Merck or Sigma-Aldrich at the highest available chemical purity.

*Separation of soluble and insoluble fractions*

The crude gum (3 g) was crushed, powdered using a mortar, wetted with ethanol (3 mL), and dispersed in water (600 mL). The mixture was kept at 8 °C overnight and then centrifuged at 5 °C and 22378 × *g* for 30 min to separate the soluble (Sol) and insoluble (Insol) parts. Both parts were freeze dried, sealed in zippered plastic bags, and stored at -20 °C for future analysis (18).

*Rheological measurements*

Concentrations of 1% and 2% crude gum, including soluble and insoluble fractions, were prepared and kept for 24 h in a refrigerator to completely disperse. The viscosity of the samples was measured using a cone and plate rheometer (C/S Plus, Brookfield, USA) equipped with temperature controlling Peltier with a shear rate increase from 0 to 400 sec^-1^ and vice versa. For this purpose, a C 50-1 spindle was used and the gap distance was adjusted to 0.5 mm. The viscosity of the samples was measured at 7, 27, and 37 °C with and without the presence of NaCl (1%). 

The rheological data were fitted to a power law model and the system consistencies (k) and power law exponents (*n*) were calculated. All experiments were performed in triplicates.

*Swelling behavior*

Tablets containing 50% of the crude gum (CG), its soluble or insoluble fractions and 50% lactose (200 ± 10 mg) were prepared using a single punch Tablet press machine (Erweka, Germany). Three Tablets of each compound were placed in 50 mL of 50 mm phosphate buffer (pH 6.8) and at different time intervals (30, 60, 90, 180, 240, 300 and 360 min) the tablets were removed, dried using tissue paper and weighed. The swelling indices (% swelling) were calculated and the average values plotted versus time (min). All experiments were performed in triplicates. The percentage of swelling at each time point was calculated as:

[(*Weight of tablet at each time*) (*Initial weight of tablet*)/ (*Initial weight of tablet*)] ×100

*Mucoadhesion properties*

Mucoadhesion of samples were determined using Texture Analyzer (CT3, Brookfield). Two cuts of cow’s tongue mucosa with 3 in 3 cm dimensions were used. For this purpose, 2% dispersions of soluble, insoluble and crude gum were prepared in deionized water and 1 mL of each sample was spread evenly on the mucosa. The values of tension forces required for detaching the surfaces were measured and presented as peak load (g) and work of adhesion (mJ). The measurements were carried out in triplet at room temperature. 

*pH measurements*

The pH of 1% concentration of crude gum, its soluble or insoluble fractions was measured using a digital pH meter in triplicate and the average was reported. 

*Isolation and characterization of carbohydrate biopolymers*

The soluble fraction (Sol) of the gum (500 mg) was applied to an ion exchange column (25 × 2 cm) packed with Sephadex A-25 which was equilibrated to pH 6.8 using NaH_2_PO_4_-Na_2_HPO_4_ 50 mM buffer and eluted with gradient solutions of 0.02, 0.05, 0.1, 0.3, 0.5, 1, 1.5, 2 M NaCl. Fractions of 8 mL were collected. Same fractions were pooled and freeze dried. Each fraction was applied to a size exclusion glass column (90 × 2 cm) packed with Sepharose CL-6B as the stationary phase and eluted with 600 mL distilled water. The isolated biopolymers were lyophilized and stored at -20 °C (19).

*Structure elucidation of biopolymers*

The protein content of the biopolymers was evaluated by Bradford assay and the uronic content determined spectrophotometrically against standard galacturonic acid. To determine the neutral monosaccharide content, 500 µg of each isolated polysaccharide was first hydrolyzed with trifluoroacetic acid (2 m) at 121 °C for 2 h and then the derivatized with N, O bis (trimethylsilyl) acetamide. The monosaccharide content was analyzed using a GC-MS instrument (Hewlett Packard 6890, USA). The gas chromatograph was equipped with a HP-5MS capillary column (Hewlett Packard, USA) (phenyl methyl siloxane, 25 m × 0.25 mm ID). The carrier gas was helium with a flow rate of 1.0 mL/min. The mass spectrometer (Hewlett Packard, USA) operated in EI mode at 70 eV and the mass range was 30-600 m/z. Identification of components was based on a comparison of the retention indices and mass spectra with Wiley (275) and derivatized standards. The injector temperature was 160 °C and sample runtime was 39 min. Oven temperature was held at 90 °C, and then increased to 140 °C at a rate of 25 °C/min. The temperature was then increased to 200 °C at a rate of 5 °C/min. The temperature was held at 200 °C for 12 min, then increased to 230 °C at a rate of 10 °C/min, and held there for 7 min (20).

*Determination of molecular weight*

A mixture of standard dextrans with different molecular weights were dissolved in water and fractionated using the same size-exclusion chromatographic method for all samples. The average molecular weight (MW) of the extracted polysaccharides was calculated as MW = ∑*M*_i_*W*_i_/∑*W*_i_ (Nishitani formula) where MW is the average molecular weight of the sample, W_i_ is carbohydrate content in the sample fraction, m_i_ is the molecular weight of standard dextran in the corresponding fraction (15, 19, 20).

*Cell proliferation assay*

Jurkat cells were cultured in complete media (CM10 containing RPMI 1640 supplemented with 10% fetal calf serum, 100 U/mL penicillin and 100μg/mL streptomycin) at 37 °C in a 5% CO_2 _at 90% humidity. The cells were then seeded in 96-well flat-bottom plates (Nunc, Roskilde, Denmark) at a density of 2 × 10^4^ cells per well and 100 μL medium containing different concentrations of polysaccharide isolates of *A. myriacanthus* in CM10 medium (31.25, 62.5, 125, 250, 500 and 1000 μg/mL) was added. After 24 h 10 μL of WST-1 reagent was added to each well and the absorbance of each well was determined at 440 nm after 4 h each test was performed in triplicate; background values from wells containing media without cells were subtracted and the average values for the triplicates were calculated. Inhibition of cell proliferation was determined by calculating the relative absorption rate of treated cells to untreated cells. In this test, etoposide (20 µg/mL) was used as a positive control (15, 19).

*Statistics*

Analysis of variance (ANOVA) was used for data analysis followed by the LSD-*t* post hoc test for multiple comparisons (Computer Statistical Package, SPSS 15). The results were expressed as mean ± SD and the differences were considered statistically significant at *P* *<* 0.05. 

## Results and Discussion

*Rheological measurements*

Table 1 shows the rheological parameters of gum and its fraction sunder different conditions. As seen, at the same concentration and temperature, the insoluble fraction showed higher consistency relative to the crude gum and soluble fraction (*P*<0.001).

The consistency of all systems decreased as temperature increased (*P*<0.001), indicating that these polysaccharides show upper critical solubility temperature (UCST). The results showed that this decrease was significantly less for the insoluble fraction *(P*<0.001), indicating its less temperature sensitivity and significantly higher for the soluble fraction (*P*<0.001), indicating its high temperature sensitivity. In nearly all cases, the pseudoplasticity (shear-thinning behavior) decreased (*n* increased) as temperature increased *(P*<0.001) and this trend was more clear for 1% concentrations (Table 1). Adding NaCl (1% w/v) to the dispersions increased the system consistency significantly (*P*<0.001) and increased shear-thinning behavior (*n* becomes negative) (*P*<0.001). These results showed that electrolytes and buffers can be efficiently used to modify the viscosity and shear-thinning properties of these polymers. Also, these results show the sensitivity of these polymers to the ions commonly present in drug formulations. The rheological behavior of natural polysaccharides and the effects of variables such as temperature and ion strength have been previously reported (21-23). The positive effect of ion strength on gel consistency has been reported for low methoxylpectins. Gigli *et al*. found that the Sol/gel transition of low-methoxyl pectin gels was very sensitive to the ion strength of the medium (21). The viscoelastic properties of gel structure were developed during 8 h cure test and retained up to 60 °C. Medina* et al*. studied the rheological properties of mucilage isolated from *Opuntia ficus-indica* (L.) Mill and found that viscosity was inversely dependent on ion strength (22). They also found a significant increase in pseudoplasticity with an increase in mucilage concentration of 1% to 10% (w/w). 

The researchers studying the other species of tragacanth reported the same results regarding the temperature effect on gel consistency and shear thinning behavior (24, 25). They also found that the insoluble fraction of Astragalus gossypinus Fisch had more consistency than the soluble fraction and the soluble fraction was more sensitive to temperature, which is in agreement with the findings of the present study (26). Nevertheless, they found that for all species, the addition of NaCl significantly decreased system consistency and shear-thinning properties. These observations show the major structural and componential differences among tragacanth gums of various species.

*Swelling*

**Figure 1:**
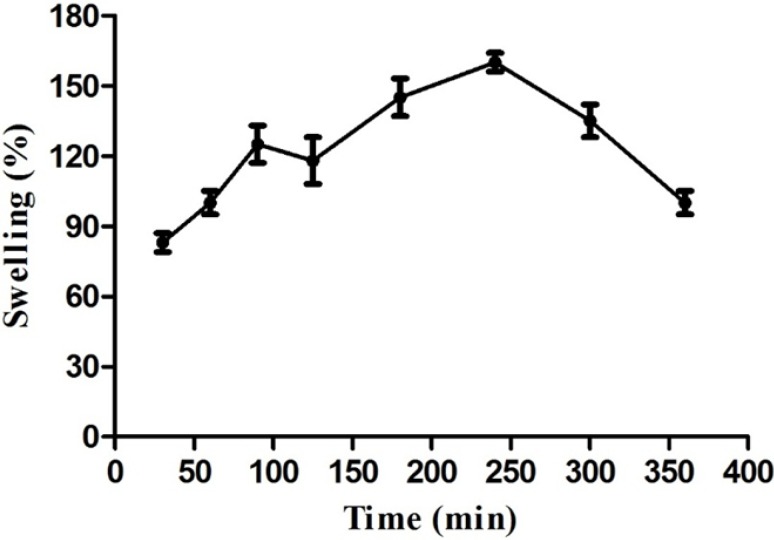
The swelling profile of *A. myriacanthus* gum versus time (*n* = 3

The tablets weights increased up to 4 h (Figure 1) due to water uptake and then decreased due to tablet erosion continued to complete erosion after 24 h (data are not shown here). Deshmukh *et al*. reported the similar swelling behavior for tablets prepared from hydrophilic gums as an excipient showing complete tablet erosion after 6 h. (27). Our study results show that gum tragacanth can swell and maintain the matrix geometry during a long time prohibiting it from disintegration which suggests it as a good candidate for controlling drug release in matrix sustained release formulations beside well-known semi-synthetic polymers such as hydroxypropyl methylcellulose (HPMC), hydroxypropyl cellulose (HPC) and sodium carboxymethylcellulose (NaCMC). In the other words, it can be considered as a natural release-modifying excipient in solid dosage form formulations.

*Mucoadhesion study*

show the mucoadhesive properties of the samples. Peak load and final load show the maximum and final forces needed to conquest the adhesion forces of samples, respectively and adhesion work presents the area under adhesion profiles. According to the results, the insoluble fraction showed higher adhesion forces compared to soluble and crude gum (*P*<0.001). Also, crude gum had higher adhesion strength than soluble fraction (*P*<0.001). This finding can be explained by structural differences between soluble and insoluble fractions of gum. Although, the researchers have studied the mucoadhesive properties of tragacanth gum and reported its superior adhesion compared to some synthetic polymers (28, 29). The current work is the first study reporting and comparing mucoadhesive properties of different fractions of tragacanth, so far.

**Table 1:** Consistency and power law model exponent for dispersions (1% and 2%) of crude gum (CG), soluble (Sol) and insoluble (Insol) fractions by temperature and presence of NaCl (*n*=3

			Consistency(K) (Pa.s)				
			7 °C	27 °C	37 °C		27 °C + NaCl
CG	1% 2%		110 ± 3.0 228 ± 4.0	98 ± 3.0 199.9 ± 3.8	45 ± 5.0 65 ± 4.0		493 ± 4. 5 939 ± 8.0
Sol	1% 2%		259 ± 4.0 353.5 ± 4.0	96.4 ± 4.5 176.5 ± 6.5	103 ± 6.1 158.1 ± 9.2		545 ± 1.5 939 ± 4.0
Insol	1% 2%		369.9 ± 5.5 571.3 ± 5.1	274 ± 6.0 381 ± 5.0	161.8 ± 4.4 277.4 ± 8.2		937 ± 3.0 1037 ± 3.0
		Power law model exponent (n)^a^					
		7 °C		27 °C	37 °C	27 °C+ NaCl	
CG	1% 2%	-0.03 ± 0.0053 0.01 ± 0.0032		0.02 ± 0.0035 0.09 ± 0.001	0.47 ± 0.151 0.10 ± 0.0086	-0.04 ± 0.0012 -0.06 ± 0.0010	
Sol	1% 2%	0.06 ± 0.0040 0.06 ± 0.012		0.08 ± 0.0070 0.02 ± 0.0033	0.49 ± 0.122 0.11 ± 0.098	-0.14 ± 0.0095 -0.04 ± 0.005	
Insol	1% 2%	-0.06 ± 0.0085 -0.02 ± 0.0042		0.04 ± 0.01 -0.03 ± 0.0032	0.13 ± 0.0087 0.05 ± 0.0097	-0.13 ± 0.0090 -0.14 ± 0.0098	

a Inversely related to the pseudoplasticity index of systems

**Table 2:** The mucoadhesion parameters of *A. myriacanthus* gum measured by Texture Analyzer (*n* = 3).

	Peak load (g)	Final load (g)	Adhesion work (mJ)
Crude gum	243.0 ± 3.7	220.6 ± 5.4	18.20 ± 0.5
Insoluble fraction	308.5 ± 5.2	266.2 ± 3.5	22.01 ± 0.5
Soluble fraction	207.4 ± 2.3	189.2 ± 2.6	15.46 ± 0.4

**Table 3:** pH values for crude gum and its soluble and insoluble fractions (*n* = 3).

	Crude gum	Soluble fraction	Insoluble fraction
pH (mean ± SD)	5.49 ± 0.04	6.14 ± 0.05	5.32 ± 0.005

**Table 4:** Molecular weight, protein content, and monosaccharide ratio of isolated soluble carbohydrate biopolymers and the insoluble fraction (Insol) of gum exudates obtained from *A. myriacanthus*

**Ratio**	**A**		**B**	**C**				**Insol**
	**A1**	**A2**	**B**	**C1**	**C2**	**C3**	**C4**	
MW(kDa)	668.36	298.08	232.42	667.8≥	667.8	539.24	58.23	_
Protein	2.77	7.51	1.47	13.41	6.41	7.88	3.65	2.02
Uronic acids	2.28	1.10	15.1	12.24	0.75	5.02	_	1.72
Arabinose	59.54	56.16	50.4	34.63	60.90	51.66	64.11	58.91
Mannose	20.17	16.09	0.39	29.58	18.50	7.96	_	1.48
Galactose	5.63	7.36	22.08	7.41	8.00	8.41	_	9.84
Glucose	1.49	4.26	_	0.93	_	18.04	29.10	7.17
Lyxose	4.98	_	4.70	_	5.40	_	_	6.58
Xylose	_	1.78	_	0.66	_	1.02	_	_
Talose	_	5.71	_	_	_	_	_	6.46
Altrose	_	_	5.86	_	_	_	_	5.58

**Figure 2.**
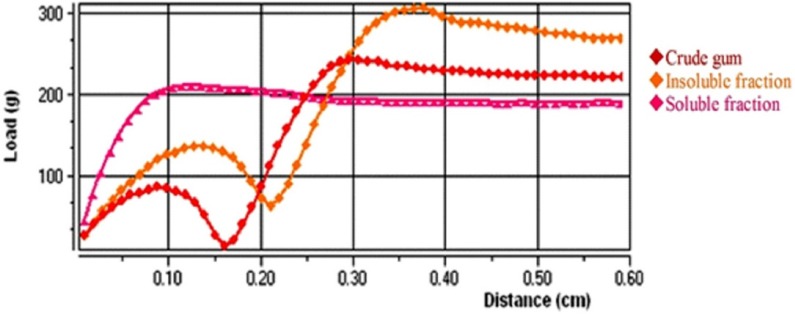
The mucoadhesive profiles of *A. myriacanthus* gum obtained by Texture Analyzer (*n* =3).

**Figure 3:**
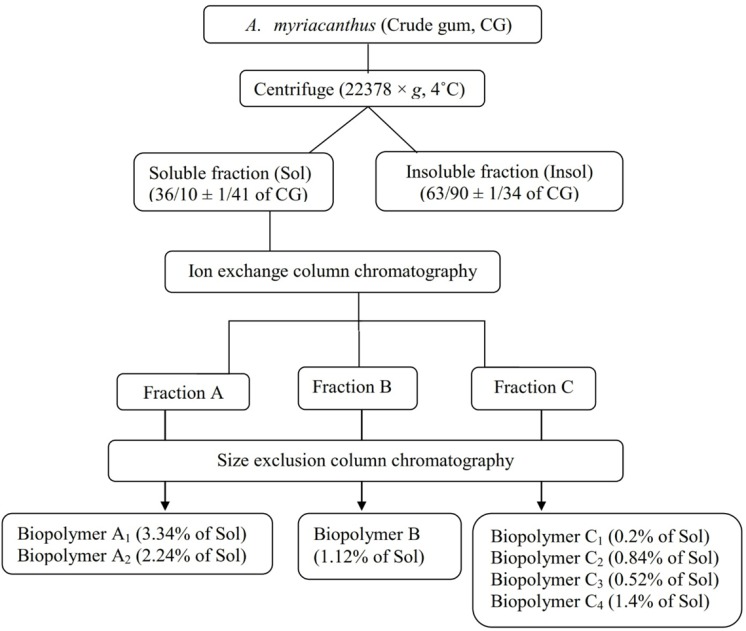
Fractionation of gum exudates obtained from *A. myriacanthus* and yields (w/w) of different isolated carbohydrate biopolymers (CG: crude gum, Sol: soluble fraction

**Figure 4.**
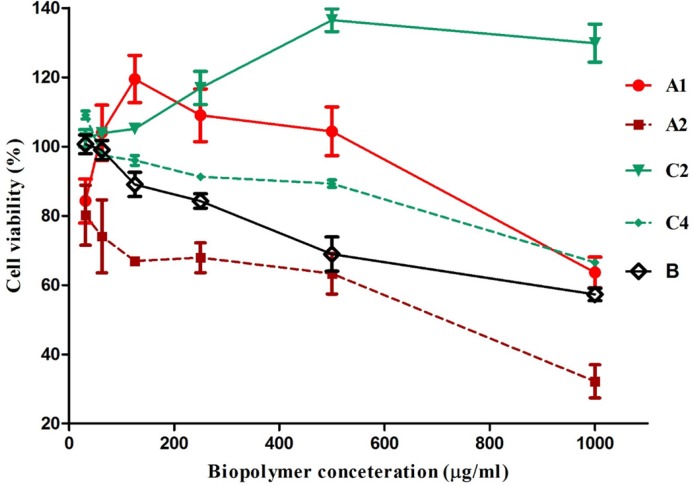
Cytotoxic/proliferative effects of isolated carbohydrate biopolymers of gum exudates obtained from *A. myriacanthus* on Jurkat cell line

*Biopolymer characterization of soluble fraction*

The soluble portion of gum tragacanth from different species of genus *Astragalus* is known as tragacanthic acid and the insoluble gel forming portion is called bassorin. Since, some misuse of these terms are found in the literature, we didn’t use them in this article,

The percentage of recovery for the soluble and insoluble fraction was 36.10 ± 1.41and 63.90 ± 1.34, respectively (Figure 3). It was assumed that the soluble/insoluble ratio (Sol/Insol) would determine the viscosity and swelling properties of tragacanth gum. Different Sol/Insol ratios have been reported for gum exudates obtained from *Astragalus* species such as 35/65 for Iranian *A gossypinus* Fisch. and Turkish *A. kurdicus *Boiss., 30/70 for Iranian *A. microcalycinus *Sirj. and Rech.f*, *65/35 for Turkish and American *A. microcephalus* Willd. and also for American *A. brachycentrus *Fisch. (25). The Sol/Insol ratio for gum exudates of this study was 

determined as 36.1/63.9

Water solubility of biopolymers is usually essential to their biological activity and bioavailability, so the water soluble part of the gum was fractionated and the structure composition and cytotoxicity of the biopolymers were evaluated. Gum tragacanth is commonly used in the food industry as a thickener and gel-forming agent because of its ability to produce a polymer-polymer matrix with proteins. These polymeric matrices are usually very sensitive to pH and electrostatic interactions (5). The pH values of the crude gum, its soluble and insoluble fractions are shown in Table 3. As seen, all pH values were acidic, which is consistent with the presence of uronic acid sugars (1.1%-15.1%) in most isolated water-soluble polysaccharides (Table 4) and the insoluble fraction (1.72%). No published reports on pH values of soluble or insoluble parts of gum tragacanth from different *Astragalus* species was found, but it has been reported that gum tragacanth in the market (usually a mixture of gums) is slightly acidic with a pH 5-6. The maximum initial viscosity of gum tragacanth is at pH 8, but the maximum stable viscosity is at about pH 5 (30).

Ion exchange chromatography was used to fractionate the biopolymers of the soluble part of the gum into 3 groups according to polarity (fractions A, B and C). The biopolymers of each fraction were isolated using size exclusion chromatography, which resulted in 7 isolated biopolymers. The recovery percentage of each biopolymer is shown in Figure 3.

Fractions A_1_, A_2_ and C_4_ were the biopolymers with the highest yields. The molecular weights of the soluble biopolymers were determined as compared to standard dextran (Table 4) and the MW ranged from 667.8 kDa for C_1_ and C_2_ to 58.23 kDa for C_4_. The Bradford assay revealed that all isolated biopolymers had protein-polysaccharide structures, but C_1_, C_3_ and A_2_ had the highest protein content (Table 4). The protein content of the insoluble fraction was 2.02 ± 0.08% (w/w).

The highest uronic acid content (15.1%) was detected for B and the C_4_ showed no acidic monosaccharides. The uronic acid content for the crude gum of six other species was calculated between 9%-37%. It was also reported that the galacturonic acid content in the gum have a significant role on stabilization of emulsions using gum tragacanth (25).

GC-MS analysis of biopolymers showed that arabinose, galactose, mannose, and glucose are major neutral monosaccharides and lyxose, xylose, mannose-6-deoxy, talose, and altrose were detected in some of these biopolymers as minor components (Table 4).

The present study is the first report on the isolation of several biopolymers from gum tragacanth, so it is not possible to make comparisons among studies. Galacturonic acid, galactose arabinose, xylose, fucose, and rhamnose from crude gum tragacanth have been reported in other species of *Astragalus*. Arabinose was reported as the major monosaccharide of the crude gum of *A. rahensis* Sirj. and Rech.f, *A. parrowianus *Boiss. and Hausskn., and *A. microcephalus* Willd. and a minor monosaccharide of *A. gossypinus* Fisch. And *A. compactus *Reiche (24, 25).

The results of this work show that most of the water soluble biopolymers have a proteoglycan structure and the polysaccharide parts of these biopolymers are arabinomannan and arabino-galactomannan (A_1_, A_2_, C_1_, and C_2_), arabinoglucan (C_3_ and C_4_), and arabinogalactan (B). Some studies have reported that branched arabinogalactan is a major component of the soluble fraction of gum tragacanth. A chain of (1-4)-linked α--galacturonic acid units with substitute β--xylopyranosyl units and terminated units of galactose or fucose was reported as the major constituent of the insoluble fraction of gum tragacanth (Balaghi *et al*. 2010). In this study, the acidic sugar content of uronic acid was determined to be 1.72% and the major monosaccharide was arabinose (58.91%). An arabinogalactan structure was also reported for a polysaccharide isolated using ethanol precipitation from an aqueous solution of gum tragacanth obtained from *A. gummifer* Labill. (30).

*Cytotoxicity/immunomodulatory assay*

Jurkat cells were used to study the immunomodulatory properties of the biopolymers. Jurkat cells are a human mature leukemic cell line which phenotypically resembles resting human T lymphocytes. They have been used to study T cell physiology in many studies (15, 19, 31). *In-vitro *cytotoxicity/proliferation effects for all the isolated biopolymers were determined, except for C_1_ and C_3_ because of their limited amount. A_2_, C_4_ and B biopolymers inhibited the proliferation of Jurkat cells in a dose-dependent manner and showed anti-proliferative activity at concentrations of 250, 500, and 1000 μg/mL. Dose-dependent enhancement of cell proliferation has been found for C_2_ at all concentrations. A_1_ isolate had an irregular effect on cell proliferation

Fattahi *et al*. evaluated the cytotoxicity of a soluble modified fraction of gum tragacanth obtained from *A. gossipinus*on on two cancerous cell lines of Hela and HepG2, and a fibroblast cell line of L929 using WST-1 assay, but did not observe significant toxicity. Slightly improved cell viability was noticed for L929 cell line (32). They modified the structure and did not isolate the polysaccharides, thus, it was possible that cytotoxic and proliferative polymers neutralized each other. Li *et al.* reported that a polysaccharide (a-(1-4)--glucan with a-(1-6)-linked branches) isolated from roots of a species of *Astragalus* (possibly Chinese *A. membranaceus*) had a proliferative effect on spleen lymphocytes in rats with gastric cancer (33).

A_1_, A_2_ and C_2_ had arabinomannan structures but each had a different effect on Jurkat cells. This indicates that other parameters beside polysaccharide structure such as molecular weight, protein and uronic acid content, monosaccharide content, and type of bond may affect immunological properties. The presence of carbohydrate-recognizing receptors on the surface of the cells may explain the biological activities of polysaccharides. For example, alveolar macrophages and L1210 mouse leukemia cells specifically bind to galactose/N-acetyl β-galactosamine, α-mannose, and α-fucose (34, 35). It has also been reported that fucose-containing carbohydrates induce differentiation of normal human keratinocytes, but no effect was observed for these carbohydrates on these cell proliferation and viability (36).

Reports on the immunomodulatory properties of arabinomannan obtained from *Mycobacterium tuberculosis* indicate that it inhibited human T cell proliferation (37, 38). The A_2_ polymer with an arabinomannan structure also decreased cell proliferation. The same type of cell was used in both studies, so the arabinomannan structure may be an important parameter in the A_2_ inhibition effect.

## Conclusion

The results of this study showed that *A. myriacanthus* crude gum and its fractions have suitable rheological properties in terms of swelling, consistency, and shear-thinning behavior. In addition, they possess a temperature-sensitive rheological behavior in physiological range which is highly interested in temperature-responsive drug delivery systems. Also, the mucoadhesion results showed that the insoluble fraction of gum is especially an excellent mucoadhesive polymer making it a suitable excipient for designing conventional and modified-release drug dosage forms. On the other hand, the soluble fraction of gum was observed to be consisted of several biopolymers (mostly proteoglycans) of different molecular weights, protein and monosaccharide contents, which differed in their immunomodulatory properties. The obtained results suggest more intensive experiments to characterize the biological and pharmacological properties of isolated carbohydrate biopolymers of gum tragacanth and not relying on the crude gum alone. 

## References

[1] Frodin DG (2004). History and concepts of big plant genera. Taxon..

[2] Jani GK, Shah DP, Prajapati V, Jain V (2009). Gums and mucilages: versatile excipients for pharmaceutical formulations. Asian. J. Pharm. Sci..

[3] Verbeken D, Dierckx S, Dewettinck K (2003). Exudate gums: occurrence, production, and applications. Appl. Microbiol. Biotechnol..

[4] Azarikia F, Abbasi S (2010). On the stabilization mechanism of Doogh (Iranian yoghurt drink) by gum tragacanth. Food Hydrocoll..

[5] Gorji EG, Mohammadifar MA, Ezzatpanah H (2011). Influence of gum tragacanth, Astragalus gossypinus, addition on stability of nonfat Doogh, an Iranian fermented milk drink. Int. J. Dairy. Technol..

[6] Nejatian M, Hatami M, Mohammadifar MA (2013). Effect of gum tragacanth exuded by three Iranian Astragalus on mixed milk protein system during acid gelation. Int. J. Biol. Macromol..

[7] Mohammadifar MA, Musavi SM, Kiumarsi A, Williams PA (2006). Solution properties of targacanthin (water-soluble part of gum tragacanth exudate from Astragalus gossypinus). Int. J. Bio. Macromol..

[8] Ramberg JE, Nelson ED, Sinnott RA (2010). Immunomodulatory dietary polysaccharides: a systematic review of the literature. Nutr. J..

[9] Tungland B, Meyer D (2002). Nondigestible Oligo-and Polysaccharides (Dietary Fiber): Their Physiology and Role in Human Health and Food. Compr. Rev. Food. SciF..

[10] Chen H, Zhang M, Qu Z, Xie B (2008). Antioxidant activities of different fractions of polysaccharide conjugates from green tea (Camellia Sinensis). Food Chem..

[11] Mestechkina N, Shcherbukhin V (2010). Sulfated polysaccharides and their anticoagulant activity: A review. Appl. Biochem. Micro..

[12] Li H, Zhang M, Ma G (2010). Hypolipidemic effect of the polysaccharide from Pholiota nameko. Nutrition.

[13] Kanekiyo K, Lee J-B, Hayashi K, Takenaka H, Hayakawa Y, Endo S, Hayashi T (2005). Isolation of an Antiviral Polysaccharide, Nostoflan, from a Terrestrial Cyanobacterium, Nostoc f lagelliforme. J. Nat. Prod..

[14] Ananthi S, Raghavendran HRB, Sunil AG, Gayathri V, Ramakrishnan G, Vasanthi HR (2010). In-vitro antioxidant and in-vivo anti-inflammatory potential of crude polysaccharide from Turbinaria ornata (Marine Brown Alga). Food Chem. Toxicol..

[15] Hamedi A, Farjadian S, Karami MR (2015). Immunomodulatory properties of Trehala manna decoction and its isolated carbohydrate macromolecules. J. Ethnopharmacol..

[16] Zippel J, Deters A, Pappai D, Hensel A (2009). A high molecular arabinogalactan from Ribes nigrum L influence on cell physiology of human skin fibroblasts and keratinocytes and internalization into cells via endosomal transport. Carbohydr. Res..

[17] Zhang M, Cui S, Cheung P, Wang Q (2007). Antitumor polysaccharides from mushrooms: a review on their isolation process, structural characteristics and antitumor activity. Trends. Food. Scie. Tech..

[18] Hamedi A, Ghanati F, Vahidi H (2012). Study on the effects of different culture conditions on the morphology of Agaricus blazei and the relationship between morphology and biomass or EPS production. Ann. Microbiol..

[19] Hamedi A, Farjadian S, Karami MR (2015). Immunomodulatory properties of Taranjebin (Camel’s Thorn) manna and its isolated carbohydrate macromolecules. J. Evid. Based. Complementary. Altern. Med..

[20] Hamedi A, Rezaei H, Azarpira N, Jafarpour M, Ahmadi F (2016). Effects of Malva sylvestris and its isolated polysaccharide on experimental ulcerative colitis in Rats. J. Evid. Based. Complementary. Altern. Med..

[21] Gigli J, Garnier C, Piazza L (2009). Rheological behaviour of low-methoxyl pectin gels over an extended frequency window. Food Hydrocolloid.

[22] Medina-Torres L, Brito-De La Fuente E, Torrestiana-Sanchez B, Katthain R (2000). Rheological properties of the mucilage gum (Opuntia ficus indica). Food Hydrocolloid..

[23] Mills PL, Kokini JL (1984). Comparison of steady shear and dynamic viscoelastic properties of guar and karaya gums. J. Food. Sci..

[24] Balaghi S, Mohammadifar MA, Zargaraan A (2010). Physicochemical and rheological characterization of gum tragacanth exudates from six species of Iranian Astragalus. Food Biophy..

[25] Balaghi S, Mohammadifar MA, Zargaraan A, Gavlighi HA, Mohammadi M (2011). Compositional analysis and rheological characterization of gum tragacanth exudates from six species of Iranian Astragalus. Food Hydrocolloid..

[26] Mohammadifar MA, Musavi SM, Kiumarsi A, Williams PA (2006). Solution properties of targacanthin (water-soluble part of gum tragacanth exudate from Astragalus gossypinus). Int. J. Biol. Macromol..

[27] Deshmukh VN, Singh SP, Sakarkar DM (2009). Formulation and evaluation of sustained release metoprolol succinate tablet using hydrophilic gums as release modifiers. Int. J. Pharm. Tech. Res..

[28] Singla AP, Goyal S, Goswami DS (2010). Characterization of mucoadhesive tablets of ciprofloxacin. WebmedCentral Pharm. Sci..

[29] Sankar GD, Sandeep G, Deepak G, Rini S, Naveen M, Kumari P, Pramod KS (2011). Formulation and evaluation of Mucoadhesive tablets of Famotidine. JPBMS..

[30] Casadeu E, Chikamai B, Singh B (2010). Gums, resins and waxes. Industrial Crops and Uses.

[31] Noorizadeh M, Hajati J (2004). Production And Partial Purification Of IL-2 Cells From Jurcat Cell Line Culture. TUMJ..

[32] Fattahi A, Petrini P, Munarin F, Shokoohinia Y, Golozar MA, Varshosaz J, Tanzi MC (2013). Polysaccharides derived from tragacanth as biocompatible polymers and Gels. J. Appl. Polym. Sci..

[33] Li R, Chen W, Wang W, Tian W, Zhang X (2009). Extraction, characterization of Astragalus polysaccharides and its immune modulating activities in rats with gastric cancer. Carbohyd. Polym..

[34] Cho C, Hoshiba T, Harada I, Akaike T (2007). Regulation of hepatocyte behaviors by galactose-carrying polymers through receptor-mediated mechanism. React. Funct. Polym..

[35] Cho C, Seo S, Park I, Kim S, Kim T, Hoshiba T, Harada I, Akaike T (2006). Galactose-carrying polymers as extracellular matrices for liver tissue engineering. Biomaterials..

[36] Becker DJ, Lowe JB (2003). Fucose: biosynthesis and biological function in mammals. Glycobiology.

[37] Oka H, Emori Y, Sasaki H, Shiraishi Y, Yoshinaga K, Kurimoto T (2002). Anti-Tumor Mechanism of Z-100, an Immunomodulatory Arabinomannan Extracted from Mycobacterium tuberculosis Strain Aoyama B, on Pulmonary Metastases of B16 F10 Melanoma: Restoration of Helper T Cell Responses via Suppression of Glucocorticoid-Genesis. Microbiol. Immunol..

[38] Oka H, Emori Y, Sasaki H, Shiraishi Y, Yoshinaga K, Kurimoto T (2002). Anti-Tumor Mechanism of Z-100, an Immunomodulatory Arabinomannan Extracted from Mycobacterium tuberculosis Strain Aoyama B, on Pulmonary Metastases of B16F10 Melanoma: Restoration of Helper T Cell Responses via Suppression of Glucocorticoid-Genesis. Microbiol. Immunol..

